# [5-(Pyridin-2-yl)-1*H*-tetra­zole-κ^2^ *N* ^4^,*N* ^5^]bis(triphenyl­phosphane-κ*P*)copper(I) tetra­fluoridoborate

**DOI:** 10.1107/S1600536812047605

**Published:** 2012-11-28

**Authors:** Lei Lu, Ping Yang, Bing Li, Lin-Fang Shi, Hua-Ru Cao

**Affiliations:** a College of Sciences, Zhejiang A & F University, Hangzhou 311300, People’s Republic of China

## Abstract

In the title Cu^I^ compound, [Cu(C_6_H_5_N_5_)(C_18_H_15_P)_2_]BF_4_, the Cu^I^ cation is *N*,*N*′-chelated by a 5-(pyridin-2-yl)-1*H*-tetra­zole ligand and coordinated by two triphenyl­phosphane ligands in a distorted tetra­hedral geometry. The tetra­zole and pyridine rings are essentially coplanar [dihedral angle = 4.1 (3)°]. The tetra­fluoridoborate anion links to the complex cation *via* an N—H⋯F hydrogen bond.

## Related literature

For applications of Cu^I^ complexes, see: Jia *et al.* (2005 ▶); Tsuboyama *et al.* (2007 ▶); Zhang *et al.* (2004 ▶). For the synthesis, see: Kuang *et al.* (2002 ▶); Demko & Sharpless (2001 ▶).
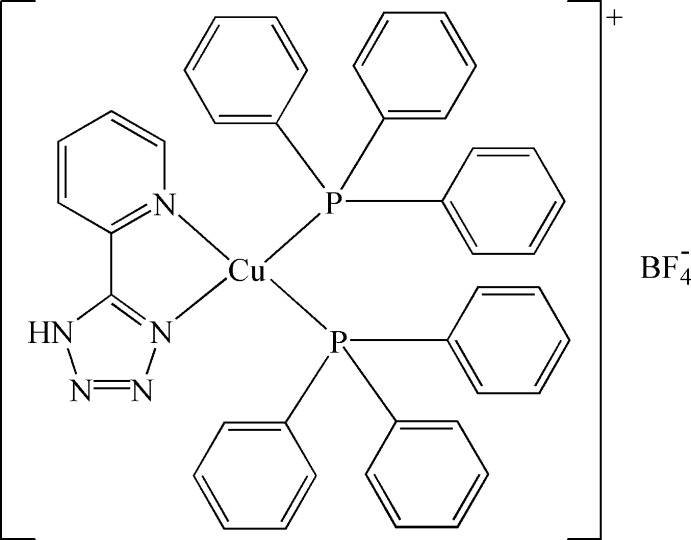


## Experimental

### 

#### Crystal data

[Cu(C_6_H_5_N_5_)(C_18_H_15_P)_2_]BF_4_
*M* *_r_* = 822.05
Triclinic, 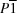

*a* = 9.6640 (19) Å
*b* = 13.052 (3) Å
*c* = 15.947 (3) Å
α = 88.66 (3)°
β = 84.80 (3)°
γ = 85.72 (3)°
*V* = 1997.3 (7) Å^3^
*Z* = 2
Mo *K*α radiation
μ = 0.68 mm^−1^
*T* = 293 K
0.29 × 0.17 × 0.16 mm

#### Data collection

Bruker SMART 1000 CCD area-detector diffractometer
Absorption correction: multi-scan (*SADABS*; Bruker, 2001 ▶) *T*_min_ = 0.908, *T*_max_ = 0.947
19069 measured reflections
8838 independent reflections
4984 reflections with *I* > 2σ(*I*)
*R*_int_ = 0.036

#### Refinement

*R*[*F* ^2^ > 2σ(*F* ^2^)] = 0.050
*wR*(*F* ^2^) = 0.166
*S* = 1.14
8838 reflections
496 parameters
H-atom parameters constrained
Δρ_max_ = 0.70 e Å^−3^
Δρ_min_ = −1.12 e Å^−3^

### 

Data collection: *SMART* (Bruker, 2007 ▶); cell refinement: *SAINT* (Bruker, 2007 ▶); data reduction: *SAINT*; program(s) used to solve structure: *SHELXTL* (Sheldrick, 2008 ▶); program(s) used to refine structure: *SHELXTL*; molecular graphics: *SHELXTL*; software used to prepare material for publication: *SHELXTL*.

## Supplementary Material

Crystal structure: contains datablock(s) global, I. DOI: 10.1107/S1600536812047605/xu5633sup1.cif

Structure factors: contains datablock(s) I. DOI: 10.1107/S1600536812047605/xu5633Isup2.hkl

Additional supplementary materials:  crystallographic information; 3D view; checkCIF report

## Figures and Tables

**Table 1:** Selected bond lengths (Å)

Cu—P1	2.2575 (13)
Cu—P2	2.2538 (14)
Cu—N1	2.185 (4)
Cu—N2	2.103 (4)

**Table 2:** Hydrogen-bond geometry (Å, °)

*D*—H⋯*A*	*D*—H	H⋯*A*	*D*⋯*A*	*D*—H⋯*A*
N5—H55⋯F4^i^	0.86	1.80	2.650 (7)	168

Symmetry code: (i) 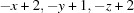.

**Fig. 1.**
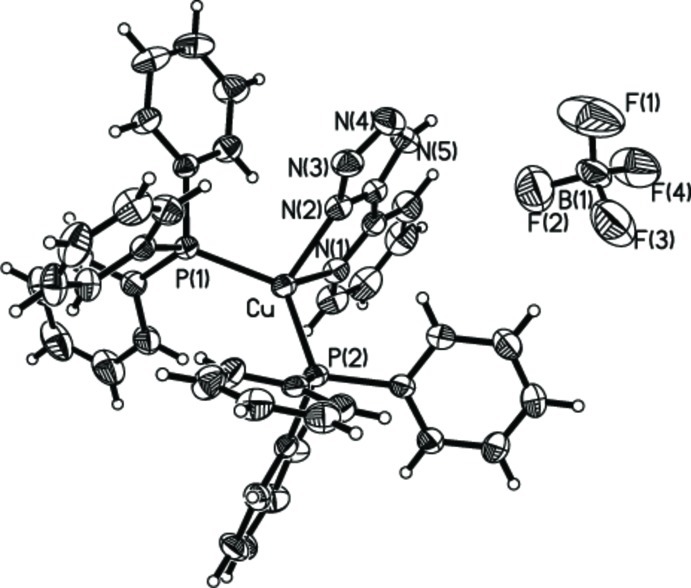
The asymmetric unit of [Cu(PPh3)2(L)]BF4, with the atomic numbering scheme. Displacement ellipsoids are drawn at the 30% probability level (arbitrary spheres for the H atoms).

## supplementary crystallographic information

### Comment

Many copper(I) complexes have been utilized in solar energy conversion, biological probing, and organic light-emitting devices (Jia *et al.*, 2005; Tsuboyama *et al.*, 2007; Zhang *et al.*, 2004). Therefore, it is pressing to explore new Cu(I) complexes served as luminescent materials. In this article, we have successfully synthesized a novel mixed ligand Cu(I) complex.

Scheme 1 and Figure 1 display the four-coordinated environment of complex [Cu(PPh_3_)_2_(*L*)]BF_4_, the coordination geometry at the Cu atom is a distorted tetrahedron. The distances of N1 and N2 to Cu1 are 2.185 (4), and 2.103 (4) Å, respectively, and the Cu—P bond lengths are 2.2575 (13) and 2.2538 (14) Å. The counter tetrafluoroboronate ion links with the complex cation via N—H···F hydrogen bonds (Table 1).

### Experimental

The 5-(2-Pyridyl)tetrazole ligand was synthesized according to the literature method (Demko & Sharpless, 2001) with some minor modification. The specific synthetic procedure is as follows: (i) To a 100 ml round-bottomed flask was added 2-cyanopyridine (0.52 g, 5 mmol), sodium azide (0.36 g, 5.5 mmol), zinc bromide (1.15 g, 5 mmol), and water (30 ml). The reaction mixture was refluxed for 5 h, cooled to room temperature. Then the mixture was basified by addition of 2.5 equiv of NaOH, filtered, acidified to pH = 1, and filtered, and the solid was washed with water then 5-(2-Pyridyl)tetrazole (0.58 g, 78%) was obtained.

[Cu(PPh_3_)_2_(*L*)]BF_4_ was synthesized according to the following procedure (Kuang *et al.*, 2002): To a 100 ml flask was added [Cu(CH_3_CN)_4_]BF_4_ 0.314 g (1 mmol), triphenylphosphane 0.522 g(2 mmol) and 10 ml dichioromethane, kept stirring for 1 h. Then 0.148 g 5-(2-Pyridyl)tetrazole was added and stirred for another hour. After the evaporation of solvent, the product was obtained as a light green powder. Single crystals of complex [Cu(PPh_3_)_2_(*L*)]BF_4_ suitable for X-ray diffraction studies were grown from slow evaporation of a CH_2_Cl_2_ solution.

### Crystal data

**Table:**

[Cu(C_6_H_5_N_5_)(C_18_H_15_P)_2_]BF_4_	*Z* = 2
*M**_r_* = 822.05	*F*(000) = 844
Triclinic, *P*1	*D*_x_ = 1.367 Mg m^−^^3^
Hall symbol: -P 1	Mo *K*α radiation, λ = 0.71073 Å
*a* = 9.6640 (19) Å	Cell parameters from 6566 reflections
*b* = 13.052 (3) Å	θ = 3.0–26.0°
*c* = 15.947 (3) Å	µ = 0.68 mm^−^^1^
α = 88.66 (3)°	*T* = 293 K
β = 84.80 (3)°	Block, light green
γ = 85.72 (3)°	0.29 × 0.17 × 0.16 mm
*V* = 1997.3 (7) Å^3^	

### Data collection

**Table:**

Bruker SMART 1000 CCD area-detector diffractometer	8838 independent reflections
Radiation source: fine-focus sealed tube	4984 reflections with *I* > 2σ(*I*)
Graphite monochromator	*R*_int_ = 0.036
phi and ω scans	θ_max_ = 27.4°, θ_min_ = 3.0°
Absorption correction: multi-scan (*SADABS*; Bruker, 2001)	*h* = −12→12
*T*_min_ = 0.908, *T*_max_ = 0.947	*k* = −16→16
19069 measured reflections	*l* = −18→20

### Refinement

**Table:**

Refinement on *F*^2^	Primary atom site location: structure-invariant direct methods
Least-squares matrix: full	Secondary atom site location: difference Fourier map
*R*[*F*^2^ > 2σ(*F*^2^)] = 0.050	Hydrogen site location: inferred from neighbouring sites
*wR*(*F*^2^) = 0.166	H-atom parameters constrained
*S* = 1.14	*w* = 1/[σ^2^(*F*_o_^2^) + (0.0409*P*)^2^ + 3.0232*P*] where *P* = (*F*_o_^2^ + 2*F*_c_^2^)/3
8838 reflections	(Δ/σ)_max_ = 0.001
496 parameters	Δρ_max_ = 0.70 e Å^−^^3^
0 restraints	Δρ_min_ = −1.12 e Å^−^^3^

### Special details

**Table:**

Geometry. All e.s.d.'s (except the e.s.d. in the dihedral angle between two l.s. planes) are estimated using the full covariance matrix. The cell e.s.d.'s are taken into account individually in the estimation of e.s.d.'s in distances, angles and torsion angles; correlations between e.s.d.'s in cell parameters are only used when they are defined by crystal symmetry. An approximate (isotropic) treatment of cell e.s.d.'s is used for estimating e.s.d.'s involving l.s. planes.
Refinement. Refinement of *F*^2^ against ALL reflections. The weighted *R*-factor *wR* and goodness of fit *S* are based on *F*^2^, conventional *R*-factors *R* are based on *F*, with *F* set to zero for negative *F*^2^. The threshold expression of *F*^2^ > σ(*F*^2^) is used only for calculating *R*-factors(gt) *etc*. and is not relevant to the choice of reflections for refinement. *R*-factors based on *F*^2^ are statistically about twice as large as those based on *F*, and *R*- factors based on ALL data will be even larger.

### Fractional atomic coordinates and isotropic or equivalent isotropic displacement parameters (Å^2^)

**Table:**

	*x*	*y*	*z*	*U*_iso_*/*U*_eq_	
Cu	0.58602 (5)	0.22693 (4)	0.77067 (3)	0.04916 (17)	
P1	0.57823 (11)	0.05451 (8)	0.77005 (7)	0.0470 (3)	
P2	0.54981 (11)	0.33726 (8)	0.66269 (7)	0.0453 (3)	
N1	0.5096 (4)	0.2971 (3)	0.8908 (2)	0.0528 (9)	
N2	0.7728 (4)	0.2473 (3)	0.8243 (2)	0.0524 (9)	
N3	0.9126 (4)	0.2261 (3)	0.8087 (3)	0.0661 (11)	
N4	0.9758 (4)	0.2553 (4)	0.8710 (3)	0.0777 (13)	
N5	0.8761 (5)	0.2956 (3)	0.9278 (3)	0.0731 (12)	
H55	0.8905	0.3207	0.9754	0.088*	
B1	1.0728 (9)	0.5802 (7)	0.8609 (5)	0.094 (3)	
F1	1.1936 (6)	0.5302 (6)	0.8667 (4)	0.235 (4)	
F2	0.9726 (7)	0.5127 (4)	0.8631 (3)	0.168 (2)	
F3	1.0651 (6)	0.6349 (5)	0.7899 (3)	0.171 (2)	
F4	1.0472 (6)	0.6438 (4)	0.9270 (3)	0.167 (2)	
C1	0.6524 (5)	0.0373 (4)	0.9330 (3)	0.0623 (12)	
H1	0.5935	0.0966	0.9411	0.075*	
C2	0.6681 (4)	−0.0088 (3)	0.8555 (3)	0.0478 (10)	
C3	0.7541 (5)	−0.0988 (4)	0.8461 (3)	0.0653 (13)	
H3	0.7650	−0.1320	0.7947	0.078*	
C4	0.8237 (6)	−0.1392 (5)	0.9129 (4)	0.0845 (18)	
H4	0.8814	−0.1992	0.9060	0.101*	
C5	0.8083 (6)	−0.0915 (5)	0.9885 (4)	0.0898 (19)	
H5	0.8557	−0.1189	1.0331	0.108*	
C6	0.7236 (6)	−0.0037 (5)	0.9990 (3)	0.0853 (17)	
H6	0.7134	0.0288	1.0507	0.102*	
C7	0.8132 (5)	0.0060 (4)	0.6619 (3)	0.0669 (13)	
H7	0.8582	0.0419	0.6997	0.080*	
C8	0.8880 (6)	−0.0373 (5)	0.5923 (4)	0.0832 (17)	
H8	0.9837	−0.0320	0.5842	0.100*	
C9	0.8223 (7)	−0.0880 (5)	0.5349 (4)	0.0889 (18)	
H9	0.8734	−0.1177	0.4883	0.107*	
C10	0.6813 (7)	−0.0948 (4)	0.5462 (4)	0.0823 (17)	
H10	0.6363	−0.1272	0.5063	0.099*	
C11	0.6053 (5)	−0.0540 (4)	0.6167 (3)	0.0614 (12)	
H11	0.5098	−0.0601	0.6246	0.074*	
C12	0.6713 (4)	−0.0038 (3)	0.6757 (3)	0.0507 (10)	
C13	0.4103 (4)	0.0003 (4)	0.7760 (3)	0.0536 (11)	
C14	0.3943 (5)	−0.1031 (4)	0.7932 (3)	0.0640 (13)	
H14	0.4715	−0.1464	0.8045	0.077*	
C15	0.2646 (6)	−0.1428 (5)	0.7937 (4)	0.0849 (18)	
H15	0.2548	−0.2122	0.8052	0.102*	
C16	0.1518 (6)	−0.0788 (7)	0.7772 (5)	0.105 (2)	
H16	0.0648	−0.1049	0.7775	0.125*	
C17	0.1653 (6)	0.0231 (6)	0.7603 (5)	0.106 (2)	
H17	0.0876	0.0661	0.7493	0.128*	
C18	0.2942 (5)	0.0625 (4)	0.7594 (4)	0.0750 (16)	
H18	0.3026	0.1319	0.7475	0.090*	
C19	0.6554 (4)	0.3099 (3)	0.5644 (3)	0.0454 (10)	
C20	0.6671 (5)	0.2101 (4)	0.5339 (3)	0.0629 (12)	
H20	0.6228	0.1588	0.5650	0.076*	
C21	0.7436 (6)	0.1857 (4)	0.4581 (3)	0.0749 (15)	
H21	0.7480	0.1191	0.4380	0.090*	
C22	0.8129 (5)	0.2602 (5)	0.4129 (3)	0.0721 (14)	
H22	0.8659	0.2437	0.3628	0.087*	
C23	0.8038 (5)	0.3585 (4)	0.4416 (3)	0.0673 (13)	
H23	0.8504	0.4089	0.4110	0.081*	
C24	0.7246 (5)	0.3833 (4)	0.5170 (3)	0.0569 (11)	
H24	0.7184	0.4506	0.5356	0.068*	
C25	0.5866 (4)	0.4693 (3)	0.6838 (3)	0.0493 (10)	
C26	0.7149 (5)	0.4857 (4)	0.7118 (3)	0.0629 (13)	
H26	0.7758	0.4298	0.7238	0.076*	
C27	0.7529 (6)	0.5842 (4)	0.7220 (3)	0.0759 (15)	
H27	0.8396	0.5947	0.7400	0.091*	
C28	0.6622 (7)	0.6666 (4)	0.7055 (4)	0.0820 (17)	
H28	0.6879	0.7331	0.7117	0.098*	
C29	0.5351 (6)	0.6514 (4)	0.6802 (4)	0.0786 (16)	
H29	0.4735	0.7076	0.6701	0.094*	
C30	0.4964 (5)	0.5533 (4)	0.6692 (3)	0.0622 (13)	
H30	0.4090	0.5438	0.6519	0.075*	
C31	0.3718 (4)	0.3494 (3)	0.6329 (3)	0.0456 (9)	
C32	0.3376 (5)	0.3416 (3)	0.5508 (3)	0.0558 (11)	
H32	0.4079	0.3320	0.5072	0.067*	
C33	0.1983 (5)	0.3481 (4)	0.5333 (4)	0.0720 (15)	
H33	0.1759	0.3415	0.4782	0.086*	
C34	0.0941 (5)	0.3641 (4)	0.5973 (4)	0.0784 (16)	
H34	0.0013	0.3671	0.5856	0.094*	
C35	0.1268 (5)	0.3756 (5)	0.6780 (4)	0.0801 (16)	
H35	0.0563	0.3895	0.7208	0.096*	
C36	0.2637 (5)	0.3666 (4)	0.6959 (3)	0.0695 (14)	
H36	0.2846	0.3722	0.7514	0.083*	
C37	0.3794 (5)	0.3180 (4)	0.9229 (3)	0.0710 (14)	
H37	0.3071	0.3033	0.8913	0.085*	
C38	0.3466 (7)	0.3612 (5)	1.0023 (4)	0.0869 (18)	
H38	0.2542	0.3763	1.0225	0.104*	
C39	0.4517 (8)	0.3807 (5)	1.0494 (4)	0.0923 (19)	
H39	0.4319	0.4080	1.1029	0.111*	
C40	0.5873 (7)	0.3598 (4)	1.0173 (3)	0.0787 (16)	
H40	0.6606	0.3735	1.0483	0.094*	
C41	0.6128 (5)	0.3183 (3)	0.9383 (3)	0.0539 (11)	
C42	0.7515 (5)	0.2904 (3)	0.8988 (3)	0.0533 (11)	

### Atomic displacement parameters (Å^2^)

**Table:**

	*U*^11^	*U*^22^	*U*^33^	*U*^12^	*U*^13^	*U*^23^
Cu	0.0538 (3)	0.0448 (3)	0.0488 (3)	0.0032 (2)	−0.0101 (2)	0.0002 (2)
P1	0.0445 (6)	0.0425 (6)	0.0540 (7)	0.0010 (4)	−0.0083 (5)	−0.0014 (5)
P2	0.0472 (6)	0.0423 (6)	0.0463 (6)	0.0039 (4)	−0.0101 (5)	0.0008 (5)
N1	0.058 (2)	0.052 (2)	0.047 (2)	0.0029 (17)	0.0012 (17)	0.0002 (17)
N2	0.052 (2)	0.051 (2)	0.052 (2)	0.0000 (16)	−0.0021 (17)	0.0046 (17)
N3	0.049 (2)	0.070 (3)	0.079 (3)	0.0067 (19)	−0.008 (2)	0.001 (2)
N4	0.057 (3)	0.085 (3)	0.094 (4)	0.002 (2)	−0.023 (3)	−0.006 (3)
N5	0.073 (3)	0.075 (3)	0.075 (3)	−0.002 (2)	−0.032 (2)	−0.009 (2)
B1	0.101 (6)	0.097 (6)	0.091 (6)	0.010 (5)	−0.057 (5)	−0.029 (5)
F1	0.174 (5)	0.282 (8)	0.247 (7)	0.130 (5)	−0.103 (5)	−0.132 (6)
F2	0.234 (6)	0.144 (4)	0.143 (4)	−0.071 (4)	−0.069 (4)	0.022 (3)
F3	0.189 (5)	0.211 (6)	0.134 (4)	−0.100 (4)	−0.066 (4)	0.044 (4)
F4	0.192 (5)	0.168 (4)	0.145 (4)	0.061 (4)	−0.079 (4)	−0.090 (4)
C1	0.069 (3)	0.062 (3)	0.054 (3)	−0.001 (2)	−0.001 (2)	0.006 (2)
C2	0.042 (2)	0.046 (2)	0.055 (3)	−0.0010 (18)	−0.0031 (18)	0.004 (2)
C3	0.068 (3)	0.056 (3)	0.070 (3)	0.010 (2)	−0.009 (3)	0.005 (2)
C4	0.077 (4)	0.081 (4)	0.090 (4)	0.022 (3)	−0.007 (3)	0.029 (3)
C5	0.085 (4)	0.116 (5)	0.067 (4)	0.007 (4)	−0.019 (3)	0.038 (4)
C6	0.104 (5)	0.099 (5)	0.052 (3)	0.004 (4)	−0.015 (3)	0.012 (3)
C7	0.052 (3)	0.082 (4)	0.066 (3)	−0.003 (2)	−0.003 (2)	−0.002 (3)
C8	0.064 (3)	0.095 (5)	0.084 (4)	0.008 (3)	0.019 (3)	−0.003 (3)
C9	0.108 (5)	0.070 (4)	0.082 (4)	−0.003 (3)	0.032 (4)	−0.013 (3)
C10	0.112 (5)	0.068 (4)	0.066 (4)	−0.023 (3)	0.011 (3)	−0.023 (3)
C11	0.065 (3)	0.056 (3)	0.063 (3)	−0.009 (2)	0.003 (2)	−0.010 (2)
C12	0.052 (2)	0.042 (2)	0.058 (3)	−0.0024 (18)	−0.006 (2)	0.005 (2)
C13	0.047 (2)	0.057 (3)	0.056 (3)	0.008 (2)	−0.005 (2)	−0.014 (2)
C14	0.054 (3)	0.065 (3)	0.073 (3)	−0.008 (2)	0.003 (2)	−0.010 (3)
C15	0.070 (4)	0.092 (4)	0.095 (4)	−0.035 (3)	0.011 (3)	−0.022 (3)
C16	0.050 (3)	0.145 (7)	0.121 (6)	−0.023 (4)	0.006 (3)	−0.055 (5)
C17	0.046 (3)	0.123 (6)	0.151 (7)	0.013 (3)	−0.022 (3)	−0.056 (5)
C18	0.052 (3)	0.069 (3)	0.107 (4)	0.006 (2)	−0.021 (3)	−0.024 (3)
C19	0.041 (2)	0.046 (2)	0.049 (2)	0.0042 (17)	−0.0122 (18)	0.0079 (19)
C20	0.074 (3)	0.047 (3)	0.065 (3)	0.007 (2)	−0.001 (2)	0.000 (2)
C21	0.096 (4)	0.060 (3)	0.064 (3)	0.014 (3)	0.001 (3)	−0.009 (3)
C22	0.070 (3)	0.084 (4)	0.059 (3)	0.015 (3)	0.000 (3)	−0.005 (3)
C23	0.063 (3)	0.077 (4)	0.060 (3)	−0.001 (3)	−0.003 (2)	0.007 (3)
C24	0.064 (3)	0.057 (3)	0.049 (3)	0.002 (2)	−0.005 (2)	−0.002 (2)
C25	0.052 (2)	0.050 (3)	0.047 (2)	0.0005 (19)	−0.0151 (19)	−0.0027 (19)
C26	0.060 (3)	0.062 (3)	0.070 (3)	−0.003 (2)	−0.023 (2)	0.005 (2)
C27	0.080 (4)	0.079 (4)	0.074 (4)	−0.022 (3)	−0.027 (3)	−0.002 (3)
C28	0.104 (5)	0.059 (3)	0.089 (4)	−0.014 (3)	−0.029 (3)	−0.020 (3)
C29	0.097 (4)	0.046 (3)	0.094 (4)	0.011 (3)	−0.026 (3)	−0.017 (3)
C30	0.065 (3)	0.052 (3)	0.071 (3)	0.007 (2)	−0.023 (2)	−0.011 (2)
C31	0.048 (2)	0.036 (2)	0.051 (2)	0.0021 (17)	−0.0053 (19)	0.0008 (18)
C32	0.060 (3)	0.056 (3)	0.052 (3)	0.001 (2)	−0.009 (2)	−0.010 (2)
C33	0.060 (3)	0.080 (4)	0.081 (4)	−0.001 (3)	−0.032 (3)	−0.017 (3)
C34	0.048 (3)	0.086 (4)	0.105 (5)	−0.009 (3)	−0.023 (3)	0.000 (3)
C35	0.048 (3)	0.102 (5)	0.087 (4)	0.002 (3)	0.001 (3)	0.015 (3)
C36	0.055 (3)	0.095 (4)	0.057 (3)	0.006 (3)	−0.005 (2)	0.008 (3)
C37	0.056 (3)	0.081 (4)	0.071 (3)	0.003 (3)	0.011 (2)	0.000 (3)
C38	0.087 (4)	0.080 (4)	0.085 (4)	0.011 (3)	0.027 (3)	0.002 (3)
C39	0.124 (6)	0.085 (5)	0.063 (4)	0.006 (4)	0.013 (4)	−0.014 (3)
C40	0.103 (4)	0.076 (4)	0.056 (3)	0.000 (3)	−0.004 (3)	−0.013 (3)
C41	0.071 (3)	0.044 (3)	0.046 (2)	0.002 (2)	−0.009 (2)	−0.0014 (19)
C42	0.059 (3)	0.045 (3)	0.057 (3)	0.000 (2)	−0.014 (2)	0.001 (2)

### Geometric parameters (Å, º)

**Table:**

Cu—P1	2.2575 (13)	C15—H15	0.9300
Cu—P2	2.2538 (14)	C16—C17	1.364 (10)
Cu—N1	2.185 (4)	C16—H16	0.9300
Cu—N2	2.103 (4)	C17—C18	1.381 (8)
P1—C13	1.812 (5)	C17—H17	0.9300
P1—C2	1.831 (4)	C18—H18	0.9300
P1—C12	1.832 (5)	C19—C24	1.381 (6)
P2—C19	1.819 (4)	C19—C20	1.394 (6)
P2—C31	1.820 (4)	C20—C21	1.389 (7)
P2—C25	1.830 (4)	C20—H20	0.9300
N1—C37	1.326 (6)	C21—C22	1.375 (7)
N1—C41	1.355 (5)	C21—H21	0.9300
N2—C42	1.320 (5)	C22—C23	1.366 (7)
N2—N3	1.360 (5)	C22—H22	0.9300
N3—N4	1.292 (6)	C23—C24	1.396 (6)
N4—N5	1.346 (6)	C23—H23	0.9300
N5—C42	1.336 (6)	C24—H24	0.9300
N5—H55	0.8600	C25—C30	1.378 (6)
B1—F1	1.304 (8)	C25—C26	1.388 (6)
B1—F3	1.329 (9)	C26—C27	1.381 (7)
B1—F4	1.351 (8)	C26—H26	0.9300
B1—F2	1.355 (9)	C27—C28	1.371 (7)
C1—C2	1.378 (6)	C27—H27	0.9300
C1—C6	1.384 (7)	C28—C29	1.357 (7)
C1—H1	0.9300	C28—H28	0.9300
C2—C3	1.390 (6)	C29—C30	1.380 (7)
C3—C4	1.384 (7)	C29—H29	0.9300
C3—H3	0.9300	C30—H30	0.9300
C4—C5	1.361 (8)	C31—C32	1.387 (6)
C4—H4	0.9300	C31—C36	1.391 (6)
C5—C6	1.362 (8)	C32—C33	1.396 (6)
C5—H5	0.9300	C32—H32	0.9300
C6—H6	0.9300	C33—C34	1.375 (7)
C7—C8	1.377 (7)	C33—H33	0.9300
C7—C12	1.384 (6)	C34—C35	1.367 (8)
C7—H7	0.9300	C34—H34	0.9300
C8—C9	1.370 (8)	C35—C36	1.375 (7)
C8—H8	0.9300	C35—H35	0.9300
C9—C10	1.367 (8)	C36—H36	0.9300
C9—H9	0.9300	C37—C38	1.399 (8)
C10—C11	1.381 (7)	C37—H37	0.9300
C10—H10	0.9300	C38—C39	1.360 (9)
C11—C12	1.385 (6)	C38—H38	0.9300
C11—H11	0.9300	C39—C40	1.373 (8)
C13—C18	1.377 (6)	C39—H39	0.9300
C13—C14	1.388 (6)	C40—C41	1.378 (6)
C14—C15	1.391 (7)	C40—H40	0.9300
C14—H14	0.9300	C41—C42	1.452 (6)
C15—C16	1.364 (9)		
			
N2—Cu—N1	78.18 (14)	C17—C16—H16	119.6
N2—Cu—P2	112.66 (11)	C15—C16—H16	119.6
N1—Cu—P2	110.72 (10)	C16—C17—C18	120.0 (6)
N2—Cu—P1	103.38 (11)	C16—C17—H17	120.0
N1—Cu—P1	114.14 (11)	C18—C17—H17	120.0
P2—Cu—P1	126.75 (5)	C13—C18—C17	120.9 (6)
C13—P1—C2	105.0 (2)	C13—C18—H18	119.5
C13—P1—C12	103.4 (2)	C17—C18—H18	119.5
C2—P1—C12	102.91 (19)	C24—C19—C20	117.5 (4)
C13—P1—Cu	119.03 (15)	C24—C19—P2	123.5 (3)
C2—P1—Cu	112.17 (15)	C20—C19—P2	119.0 (3)
C12—P1—Cu	112.77 (15)	C21—C20—C19	121.3 (5)
C19—P2—C31	104.20 (18)	C21—C20—H20	119.4
C19—P2—C25	102.9 (2)	C19—C20—H20	119.4
C31—P2—C25	103.88 (18)	C22—C21—C20	119.9 (5)
C19—P2—Cu	116.03 (13)	C22—C21—H21	120.1
C31—P2—Cu	114.80 (14)	C20—C21—H21	120.1
C25—P2—Cu	113.54 (14)	C23—C22—C21	120.0 (5)
C37—N1—C41	117.5 (4)	C23—C22—H22	120.0
C37—N1—Cu	129.1 (3)	C21—C22—H22	120.0
C41—N1—Cu	113.3 (3)	C22—C23—C24	120.1 (5)
C42—N2—N3	107.1 (4)	C22—C23—H23	120.0
C42—N2—Cu	112.5 (3)	C24—C23—H23	120.0
N3—N2—Cu	140.3 (3)	C19—C24—C23	121.3 (5)
N4—N3—N2	110.0 (4)	C19—C24—H24	119.3
N3—N4—N5	106.4 (4)	C23—C24—H24	119.3
C42—N5—N4	109.5 (4)	C30—C25—C26	118.5 (4)
C42—N5—H55	125.3	C30—C25—P2	123.3 (3)
N4—N5—H55	125.3	C26—C25—P2	118.1 (3)
F1—B1—F3	113.6 (9)	C27—C26—C25	120.6 (5)
F1—B1—F4	108.3 (5)	C27—C26—H26	119.7
F3—B1—F4	109.1 (7)	C25—C26—H26	119.7
F1—B1—F2	109.4 (8)	C28—C27—C26	119.7 (5)
F3—B1—F2	106.8 (5)	C28—C27—H27	120.2
F4—B1—F2	109.5 (8)	C26—C27—H27	120.2
C2—C1—C6	120.7 (5)	C29—C28—C27	120.2 (5)
C2—C1—H1	119.7	C29—C28—H28	119.9
C6—C1—H1	119.7	C27—C28—H28	119.9
C1—C2—C3	118.3 (4)	C28—C29—C30	120.5 (5)
C1—C2—P1	117.8 (3)	C28—C29—H29	119.7
C3—C2—P1	123.8 (4)	C30—C29—H29	119.7
C4—C3—C2	120.3 (5)	C25—C30—C29	120.4 (5)
C4—C3—H3	119.9	C25—C30—H30	119.8
C2—C3—H3	119.9	C29—C30—H30	119.8
C5—C4—C3	120.4 (5)	C32—C31—C36	118.0 (4)
C5—C4—H4	119.8	C32—C31—P2	123.6 (3)
C3—C4—H4	119.8	C36—C31—P2	118.4 (3)
C4—C5—C6	120.0 (5)	C31—C32—C33	120.3 (5)
C4—C5—H5	120.0	C31—C32—H32	119.9
C6—C5—H5	120.0	C33—C32—H32	119.9
C5—C6—C1	120.3 (6)	C34—C33—C32	120.1 (5)
C5—C6—H6	119.9	C34—C33—H33	120.0
C1—C6—H6	119.9	C32—C33—H33	120.0
C8—C7—C12	120.2 (5)	C35—C34—C33	120.1 (5)
C8—C7—H7	119.9	C35—C34—H34	119.9
C12—C7—H7	119.9	C33—C34—H34	119.9
C9—C8—C7	120.4 (5)	C34—C35—C36	120.0 (5)
C9—C8—H8	119.8	C34—C35—H35	120.0
C7—C8—H8	119.8	C36—C35—H35	120.0
C10—C9—C8	119.8 (5)	C35—C36—C31	121.4 (5)
C10—C9—H9	120.1	C35—C36—H36	119.3
C8—C9—H9	120.1	C31—C36—H36	119.3
C9—C10—C11	120.4 (5)	N1—C37—C38	122.5 (5)
C9—C10—H10	119.8	N1—C37—H37	118.7
C11—C10—H10	119.8	C38—C37—H37	118.7
C10—C11—C12	120.1 (5)	C39—C38—C37	119.1 (6)
C10—C11—H11	120.0	C39—C38—H38	120.5
C12—C11—H11	120.0	C37—C38—H38	120.5
C7—C12—C11	118.9 (4)	C38—C39—C40	119.3 (6)
C7—C12—P1	118.1 (4)	C38—C39—H39	120.3
C11—C12—P1	123.0 (3)	C40—C39—H39	120.3
C18—C13—C14	118.1 (4)	C39—C40—C41	118.8 (6)
C18—C13—P1	119.2 (4)	C39—C40—H40	120.6
C14—C13—P1	122.6 (3)	C41—C40—H40	120.6
C13—C14—C15	120.9 (5)	N1—C41—C40	122.7 (5)
C13—C14—H14	119.5	N1—C41—C42	113.5 (4)
C15—C14—H14	119.5	C40—C41—C42	123.8 (5)
C16—C15—C14	119.3 (6)	N2—C42—N5	107.1 (4)
C16—C15—H15	120.4	N2—C42—C41	122.5 (4)
C14—C15—H15	120.4	N5—C42—C41	130.3 (4)
C17—C16—C15	120.7 (6)		

### Hydrogen-bond geometry (Å, º)

**Table:**

*D*—H···*A*	*D*—H	H···*A*	*D*···*A*	*D*—H···*A*
N5—H55···F4^i^	0.86	1.80	2.650 (7)	168

Symmetry code: (i) −*x*+2, −*y*+1, −*z*+2.
